# Lectures on the Diseases of the Bones and the Joints

**Published:** 1844-10-19

**Authors:** Robert Liston

**Affiliations:** Surgeon of University College Hospital, and Professor of Clinical Surgery in University College, London


### CLINICAL LECTURES AND REPORTS.

### LECTURES ON THE DISEASES OF THE BONES AND THE JOINTS.

BY ROBERT LISTON, ESQ., F.R.S.,

Surgeon of University College Hospital, and Professor of Clinical Surgery in University College, London.

*Substitute bone after the loss of bone from death. Error relating to necrosis without sequestra. Mr. Gulliver's experiments on the disappearance of dead bone. Treatment of periostitis. The veterinary operation of subcutaneous periostotomy. Treatment of inflammation of bone. Of caries and necrosis. Removal of the sequestrum. Hypertrophy and atrophy of bone. Rickets and mollities ossium.*

When the greater part of the shaft of a long bone dies, new osseous matter forms a case around the dead bone, which case is called the "substitute bone."

The centre of the bone is, of course, filled with purulent matter; the bone itself becomes much enlarged; very intense pain is experienced; and the system in general suffers considerable disturbance. After a time, however, ulceration proceeds in some part of the encasing bone, a "cloaca" is formed, and the matter until now pent up in the medullary cavity, finds an exit into the soft parts round the bone, and points externally. As soon as the quantity of matter thus collected has escaped, and the parts become quieter, the opening in the soft parts contracts: the aperture is converted into what is called a "papilla" (which is an elevated granulation having a small hole in the centre.) The papilla leads by a narrow fistulous opening to the cloaca, and probably the probe passed through this may enable us to feel the dead bone within.

This state of parts remains, often for a long time, until the dead portion becomes separated from the living portion by the process of absorption, and during this period the "substitute bone" is being made more and more capable of carrying on the functions of the part which has been killed.

The formation of the substitute bone proceeds from those extreme points of the old bone which retain their vitality, and takes place *under* the thickened and swollen periosteum; for the bony tissue alone seems to have the power of assimilation, as it were, and of causing the deposition on its surface of new osseous matter. Although, however, it is commenced at several isolated spots, it ultimately becomes one piece by the little islands of bony matter coalescing and uniting at the points at which they touch one another.

This, as I have said, is going on together with the process of separation. Now, how does this take place? Why, the dead bone is separated from the living bone precisely in the same manner that the mortified parts are separated from the living in cutaneous, muscular, and other structures; that is to say, the part of the living bone in immediate contact with the dead portion is absorbed by the proper vessels, and the sequestrum is ultimately left free.

This absorption of the living bone takes place close around the dead piece, or pieces, so that these present irregular cellular edges covered with fine spiculæ. The appearance of erosion of the edges of sequestra has led to the idea that these, after their separation, and whilst they are lying loose in the cavity of the substitute bone, are in some degree acted on by the absorbent vessels. Nay, it has been supposed by some that if the sequestra remain long enough they are entirely removed by this process, and cases are related of necrosis without sequestra. This idea, however, is entirely erroneous, and the nature of the cases I allude to is this. A small portion of bone in the central part of a bone dies, and violent inflammation of the whole bone is the consequence, even though the exciting cause be small. This inflammatory action continues for a long time, and either the small piece of dead bone is discharged unnoticed with the pus, or it becomes encased, and ultimately lies dormant in the centre of the bone. The external opening then heals, and the case is supposed to be one in which the dead bone has been absorbed. There is no such thing as any absorption of bone after once it has become dead, and I have long insisted on this doctrine in my lectures. It is beautifully shown in this preparation of a long sequestrum, composed of nearly the whole internal shell of the humerus. The upper part of this sequestrum presented just under the shoulder, having pushed through a cloaca in that situation; and, as all of it could not be extracted, I applied the cutting forceps, and removed as much as I could reach. The opening now closed, and the sequestrum remained for a long time bathed in the pus which filled up the medullary cavity, whilst the process of absorption was slowly going on in the neighbouring living bone. At length, when separation had taken place sufficiently to allow the sequestrum to be moved about, a fresh incision was made over the shoulder, and the top of the sequestrum being seized by forceps the whole was drawn out in one piece. If you examine it you will see that, although all the rest of the sequestrum is cellular in its appearance, and has rough and apparently corroded edges, the upper part, where the bone-forceps was applied, is as smooth and sharp as when it was first cut through, clearly showing that no absorption had been going on in the actually dead bone, though it had been proceeding in the living bone in close contact with it. The same fact is illustrated in some cases of amputation. When everything goes on favourably the end of the amputated bone becomes quickly smoothed and rounded off by the absorption of the edges and asperities left by the saw; but when, as occasionally happens, the end of the bone becomes necrosed, although it may be covered over with granulations, and remain concealed in this way for a length of time, yet the cut end remains as clean and smooth as ever, and not the slightest absorption takes place around its edges.

This matter has lately been completely set at rest by my friend, Mr. Gulliver, who made several interesting experiments to discover whether or not dead bone could be acted on by the absorbents of living bone. He was told by Mr. Clift, of the College of Surgeons, that John Hunter had made some experiments on the subject, and had found that when a piece of dead bone was bound on an ulcer, it was acted on by the living structures, and partially absorbed. Other experimenters tried the same thing with some variations, and declared that they found even that ivory when so treated was partly taken up. These experiments Mr. Gulliver first performed, carefully weighing the bone before and after the experiment; and though he sometimes found the weight of the piece slightly increased, probably owing to the absorption of moisture from the ulcer, he never could discover the slightest diminution in it.

The same result has been obtained at the hospital, where I have caused small smooth pieces of bone, previously weighed, to be introduced, and kept in issues, instead of peas, or other foreign bodies. But Mr. Gulliver went farther,—he introduced pieces of dead bone into the medullary cavities of the long bones of rabbits and other animals, and, after leaving them in for several weeks, killed the animal and examined the state of parts. He never could find the bone which he had introduced in the slightest degree eroded, nor could he detect any diminution in its weight. In some cases it was curious that he observed the dead pieces of bone cemented to the sides of the medullary cavity by new osseous matter thrown out by the living bone.

So much, then, with regard to the separation of sequestra. When that has been effected the substitute bone has to perform all the duties of the bone unassisted, and generally it is quite able to do so. Sometimes it is not strong enough, and is fractured by muscular action or violence, but that is seldom the case, for, as I have before observed, it begins to be deposited even before the old bone is actually dead, and continues to increase in size whilst the process of separation is slowly going on.

The substitute bone is at first very clumsy and unsightly, having a very rough irregular surface, with large nodules projecting, and numerous cloaca in different parts. These, however, are gradually rounded off and filled up, and the medullary cavity being also diminished in calibre, the bone in time becomes very much like the original one, both in its external appearance and internal structure. In the last place, with respect to the suppuration in this disease, it is at first excessive, but gradually diminishes, although it does not quite cease until the dead bone, which keeps up irritation like any other foreign body, is removed; and although at the commencement of the disease the system suffers considerably from the copious formation of pus, the constitution appears after a time to become accustomed to the drain which is thus established. The character of the pus in necrosis is generally thick, healthy, and bland, and differs very much from the foul, fetid, and irritating discharge which escapes from that kind of ulcer in bone which I have mentioned to you under the name of *caries*.

We now come to consider generally the mode of treatment of these affections; I say generally, because I propose, after speaking of the diseases of joints (which are so intimately connected with disease of the bones as to require to be considered in relation with them,) to treat shortly of diseases of particular bones, commencing with those of the skull and spine, and proceeding to those of the extremities.

In that kind of inflammation which has its seat in the *periosteum* you must look well to the predisposing causes. Some arise from a natural vice in the constitution, others from abuse of mercury, and others from exposure to cold whilst the patient is undergoing a mercurial course; others, again, are of a perfectly simple nature, arising from injury, &c. The *general treatment* will vary in such cases according to these circumstances. In the simplest cases it will consist of the exhibition of diaphoretics, sarsaparilla, &c., and in others close attention must be paid to the general health, and to improve the tone and strength of the patient.

In the most violent cases, however, we must sometimes have recourse to mercury, even where that medicine may have been the original predisposing cause of the disease. As the method least likely to prove hurtful to the constitution I should recommend the exhibition of the *bichloride or biniodide of mercury*, which should be continued, steadily, until some effect is produced on the tumour, or until the nocturnal pains are lessened. It may then be laid aside, and in its place you may administer *decoction of sarsaparilla*, with or without the *iodide of potassium*, which in many cases gives great and sometimes permanent relief. Other medicines might be mentioned, but these are sufficient for our purpose.

The disease also requires to be pretty actively treated by *local means*. The local abstraction of blood by leeches will often be borne with great advantage; after which blisters may be applied, either in the ordinary way, or still better by rubbing the part, previously moistened, with nitrate of silver, or the skin over the inflamed periosteum may be painted over with a strong solution of iodine and alcohol (ʒj—ʒiv.)

In some cases of nodes these methods of treatment often prove unsatisfactory, and then I would be inclined to have recourse to incisions. These should be made freely down to the bone, and tolerably long. If matter have collected it is thus got rid of, and if not the bleeding which takes place is very serviceable, and the swelling and extreme tension of the parts, productive of so much pain, are relieved. I have often wished, though I have not yet accomplished my desire, to perform in these cases an operation which veterinary surgeons perform on young horses in cases of what is termed "splints." The operation to which I refer is called "subcutaneous periostotomy," and consists in making a small puncture in the skin, and introducing a probe-pointed knife, its edge on the convexity, which, when its edge is turned against the bone, cuts through the inflamed periosteum without dividing any more of the soft parts. This operation is often attended with excellent results in horses, and I do not see why it should not prove equally beneficial if applied to proper cases in the human subject. It is a very simple proceeding, and is not attended with more disturbance of the parts than is excited by division of tendons in cases of club-foot. (This operation has since been tried with good effect.)

In *inflammation of particular bones* the treatment should be conducted much on the same principle as in periostitis. It should always be prompt and generally energetic, as we can more easily prevent the many serious consequences which may ensue from it than treat them when once they occur. In acute inflammation of bones you will frequently, and indeed in most cases when the patient is tolerably strong, find it necessary to bleed copiously and perhaps repeatedly. The bowels also should be freely moved by an active purge, and means may then be taken to determine to the skin by the use of diaphoretic medicines, baths, &c. Of course the patient should at the same time be kept perfectly quiet, on a low diet, and should be protected from the influence of cold.

When suppuration follows inflammation, and that suppuration is superficial, of course the sooner the matter is let out the better for the patient, as less risk is run of further unpleasant consequences from its accumulation and pressure. When symptoms arise threatening suppuration in the heads of the long bones the limb should be entirely disused, and kept elevated, and powerful counter-irritants should be had recourse to. The moxa, and the application of the red hot iron, are prised by many as the only modes of commanding such deep-seated inflammation. At one time I used to employ the actual cautery, but I have now given it up for the potential cautery, as I consider the issues formed by the application of potassa fusa, corrosive sublimate, &c. to be equally efficacious, whilst their employment is much less appalling to the patients and their friends.

When matter collects in the medullary cavity of bones, or between the two tables of flat bones (called absurdly enough "spina ventosa") the patient suffers extreme pain and constitutional disturbance, which renders it necessary to get rid of the collection. In some parts of a bone so affected the osseous matter gives way readily, it is rapidly removed by absorption, and becomes extremely thin, almost diaphanous when dry—ultimately it may burst altogether. In other parts, however, there may be a fresh deposition of bony matter, and the shell of the bone instead of becoming thinner actually increases in thickness. In such a case it will be necessary to interfere actively, and make an opening for the exit of the pus by means of a trephine. This is not unfrequently the case in the tibia, and when I come to treat of diseases of that bone I shall have the opportunity of recurring to this subject.

When ulceration has taken place an early and free opening should be made over the part if possible, to let the matter out, and allow the parts a chance of recovering themselves. If the ulcer be of that kind which is denominated carious, such a proceeding proves useless, and the diseased bone must be removed. If there be any necrosed bone present it should be turned out with a sharp scoop (such as is used by bird-stuffers for removing the brain,) and at the same time the carious bone should be as freely removed as possible, the lardaceous and tuberculous deposit, so often present in these cases, being then likewise got rid of. After this proceeding the opening in the bone should be dressed with dry lint and allowed to granulate from the bottom. Sometimes the application of an escharotic is required to determine the death of a carious portion of bone, and cause it to be fairly thrown off. The application should be a dry one. That which I generally employ is *red oxide of mercury*. Such applications are much better and more convenient than caustic potass, or nitrate of mercury dissolved in nitric acid, &c., which spread about, and are not so limited in their action, or so precise in their effects, as could be wished.

When speaking of the treatment of caries I said that in some cases the diseased portion of bone might be removed, and the limb or part remain serviceable, but this is not very frequently the case, and you will be obliged to have recourse to a more severe proceeding. When the affection is limited to the heads of bones composing a joint, as the shoulder or elbow, for example, and the case is in other respects favourable, the patient may escape with a tolerably useful limb, by submitting to the excision of the joint, of which I shall hereafter have occasion to speak again. Often, however, this is impracticable, and amputation of the limb above the diseased part is the only chance which the patient has of regaining health and strength.

Now, with regard to the treatment of *necrosis*, when once the bone is dead you must never hope, as I have already shown you, that it can become absorbed. Hence the use of frequent blisters, tincture of iodine, and other applications, made with the view of promoting absorption, is more than doubtful. The same may be said of lotions containing acids in solution, which are by some applied over the wound, or injected into the cavity containing the sequestrum. These can never determine the absorption of the sequestrum. All these things are even worse than useless. Neither, again, can you reasonably expect that the dead portion or portions of bone will remain dormant, though sometimes this may be the case. In general, local and perhaps constitutional irritation will be kept up until the offending parts, which act like any other foreign body, are expelled or extracted; you must, therefore, endeavour to get your patient quit of the sequestrum as soon as possible. There is no use in trying to extract it by any operation until there is reason to think that the process of absorption in the neighbouring living bone has advanced to such an extent as to loosen the sequestrum. When the case is one of very long standing you may fairly suppose that this has taken place, but in general you should obtain more accurate information by the use of the probe.

You pass this instrument into a cloaca, and you find that it can be pushed between the new and the dead bone; by turning the instrument in a skilful manner you may then be able to pass it round the other side, so as to satisfy yourself that the part near the cloaca is quite separated. If the bone is so exposed that it can be laid hold of with forceps, you can then shake it and move it backwards and forwards in the direction of the shaft, or this may be done sometimes by fixing a sharp point, as of a pair of scissors, in it; thus its detachment can be determined. I have often used with advantage for this purpose a steel probe with a fine screw upon it, such a one as is used for removing tubes from the nasal duct. When you discover for certain that the bone is loose, you are warranted in cutting down upon the opening and trying to get out the dead portion of bone, even though you cannot move the piece upwards and downwards in the cavity of the substitute bone. This may, however, be done in some cases. In dividing the soft parts you generally meet with sharp hæmorrhage, as the tissues are condensed and the blood-vessels cannot easily retract. When you arrive at the opening in the substitute bone, you may find it too small to allow of the exit of the sequestrum, and will then proceed to enlarge it to the requisite extent. This is done by cutting away the new bone, which is readily accomplished in most instances. Sometimes you will find two cloaca near each other, and will only have to clip up or saw away the bone which separates the two, in order to get a sufficiently large opening. When this is accomplished you lay hold of the dead bone with a pair of strong, deeply-toothed forceps, and try to extract it. You may thus draw out a long piece in the direction of the shaft, or you may have to take out numerous pieces from the same bone. In other cases you will experience more difficulty, and will be obliged to vary the operation according to the circumstances of the case. For example, you may happen to cut down upon the dead bone near its centre, and whilst you can move it both upwards and downwards to a certain extent, you cannot free either extremity so as to draw the whole out. In such a case, common sense will tell you to apply a small saw, (Hey's straight-edged saw,) and, after cutting the sequestrum across the middle, extract each half separately. When you have removed the whole of the disease the parts are allowed to granulate and close, which they begin to do immediately. If the new bone is not strong enough, you may for a time put the limb in splints and a bandage; but, as I before said, this is very seldom required; and, for my own part, I never yet saw a case in which such a proceeding was indicated. All you have to do is to attend to the patient's general health, and nature will conclude the case for you without difficulty.

Having now taken a general view of the nature and treatment of inflammation of bone and its consequences, we have to notice two other diseases of this tissue, before we proceed to affections of the joints—I allude to hypertrophy and atrophy.

*Hypertrophy* is sometimes seen in gouty subjects affecting the bones of the wrist and other joints, giving an enlarged and awkward appearance to them, not dependent on inflammatory swelling. When such joints are examined, the articular ends of the bones are found swollen and loose in texture—the bony matter is perhaps not so dense and close as usual, but still there is more of it than natural. Again, you will occasionally meet with cases in which the articular cartilage has been removed by ulceration, and in which an excessive deposite of bony matter has taken place, forming a dense, smooth, and even polished longer on the surfaces, which are required to move over one another. This porcelainous deposite is seen in the hip-joint occasionally, and consists in hypertrophy of the tissue. In this instance (specimen) it is attended with immense deposite of bone around the joint, causing the depth of the acetabulum to be greatly increased. The "ligamentum labri cartilagineum," as it has been called, has, in fact, been completely ossified, and the head of the femur is in some positions, completely locked in. The other large joints may likewise be the seat of this unusual deposite.

The opposite state of *atrophy* is not unusual; either particular bones or the whole osseous system becoming wasted, and the true bony tissue being in part removed by interstitial absorption.

The bone is altered in its constituent parts; the shell is thinned; the cancellated structure becomes more open and bears a greater proportion than natural to the dense part; and the cells are filled with oily or fatty matter. It is specifically as well as absolutely lighter, than it was in health. This takes place in the bones of limbs which, from some obstinate disease, have been long disused; and not only is the structure of the bone altered, but its size is also diminished, particularly in circumference. Instances of local atrophy of bones are seen in an affection of the hip, worth remembering. A patient falls and strikes the hip, and you find, on examniation, that there is no fracture; that the joint is only bruised. However, there is some slight inflammatory action, and, consequently, the joint is for some time disused, owing to the pain excited on motion. The effect of this disuse may be an *interstitial absorption* of the head of the bone sufficient, ultimately, to cause as much as two inches' shortening of the limb. This *may* take place in persons of middle life, though more commonly met with in old people; and as the lameness consequent on it is as bad and as permanent as if the neck of the bone had been broken, it is a very important affection to be acquainted with. It is also seen in the whole skeleton of persons who have been bed-ridden for many years before death, as in a remarkable case in my museum of a poor hydrocephalic patient whose bones are reduced to almost half their natural dimensions.

The same kind of thing now and then occurs in bones, from a want of vascular supply, and this only in part of a bone. Thus, as Mr. Curling has lately shown, when fracture of the leg, &c., takes place below the point at which the nutritious artery enters, the bone below the fracture becomes atrophied from deficient nutrition.

Again, atrophy of the bones, as well as of the other structures, of one or more limbs, will sometimes occur in children who have been afflicted with that form of paralysis which not unfrequently follows on difficult teething attended with convulsions, &c., The limb cannot be moved by an effort of the will; it remains for a long time inactive; and as a natural result of such want of exercise, all the tissues in it become wasted, and the bones among the rest.

In some weak and unhealthy children, the bones are imperfectly formed from the commencement, and instead of becoming the seat of ossification, as in healthy subjects, they remain for a length of time soft and cartilaginous, containing a quantity of brownish bloody serosity, and very little osseous matter or true marrow. In consequence of this, when the child begins to get about, the long bones are observed to give way; the femur bends in so as to let the knees cross one another; the bones of the leg become distorted; the pelvis gives way in different parts, and progression is thereby rendered painfully difficult. After a time, also, the ribs themselves are flattened in different directions, and breathing becomes laborious. Such children are, generally, of a very weak habit and unhealthy appearance; their belly is prominent; their digestive system out of order; and their bowels, perhaps, irregular. This, however, may be recovered from, though not, perhaps, until irreparable mischief has been effected. The general health of the child is improved, and as it becomes stronger the bones harden in their distorted shapes by the gradual deposition of osseous matter. Ultimately, the osseous system becomes as dense as usual, but the individual, particularly if a female, is exposed to much inconvenience and even danger. It has been noticed that these women are peculiarly disposed to conception, and each time that such an event takes place, they are probably obliged to undergo some operation, either to bring on premature labour or to cut the child's head in pieces, in order to allow of its extraction. When this is neglected the patient is in great danger, owing to the limited space allowed for the passage of the child's head between the mis-shapen bones of the pelvis. The Cæsarian operation, and other severe proceedings, are also had recourse to in some such cases. Now, this diseased state of the bones is called rachitis or rickets. It is an original deficiency, and may be only temporary.

There is another affection, very much like rickets in its intimate nature, called mollities ossium, which differs from it, however, in not being an original defect. Persons labouring under this disease may have been perfectly formed at one time; their bones may have been straight enough, and properly composed, so far as relates to the comparative quantity of osseous matter. From some obscure cause, however, they fall into ill health, and all their bones soften till they be perfectly pliable, and may be bent about in any direction. In this state, if the bones are examined, they will be found to contain an unusually small proportion of phosphate of lime; they are, in fact, very much in the same state as a healthy bone which has been steeped for a time in some acid solution. Mollities ossium also differs from rachitis in being (at least as far as can be observed, and it is, fortunately, a rare disease) a permanent affection. The osseous matter which *has never been formed* in the case of rickets *may*, after a longer or shorter period, be deposited, and the disease so far remedied; but when it has once been deposited and then taken up by the absorbents, as in mollities ossium, the earthy matter is said never to make its appearance again, and the patient is condemned to linger out a miserable existence.

Softening of the bones has been attributed to the imprudent and excessive employment of mercury, to hæmorrhages from different causes, and to other circumstances tending to debilitate the patient. It has also been referred to the effect of common salt taken in large quantities, but I cannot understand how this should produce such an effect.

*London Lancet.*

